# Genome sequences published outside of *Standards in Genomic Sciences,* October – November 2011

**DOI:** 10.1601/sigs.2404675

**Published:** 2011-11-23

**Authors:** Oranmiyan W. Nelson, George M. Garrity

**Affiliations:** 1 Editorial Office, Standards in Genomic Sciences and Department of Microbiology, Michigan State University, East Lansing, MI, USA

## Abstract

The purpose of this table is to provide the community with a citable record of publications of ongoing genome sequencing projects that have led to a publication in the scientific literature. While our goal is to make the list complete, there is no guarantee that we may have omitted one or more publications appearing in this time frame. Readers and authors who wish to have publications added to subsequent versions of this list are invited to provide the bibliographic data for such references to the SIGS editorial office.

**Phylum** *Crenarchaeota*
*Thermoproteus tenax*, strain Kra1, DSM 2078^T^ sequence accession FN869859 [1]
**Phylum** *Euryarchaeota*
*Haloarcula hispanica* CGMCC 1.2049, sequence accession CP002921 (chromosome I), CP002922 (chromosome II), and CP002923 (plasmid pHH400) [2]
*Methanococcus maripaludis*, strain X1 (unculturable) sequence accession CP002913 [3]
**Phylum** *Proteobacteria*
*Acinetobacter baumannii* strain 1656-2, sequence accession CP001921 [4]
*Arcobacter butzleri* strain ED-1, sequence accession AP012047, AP012048, and AP012049 [5]
*Brucella suis* strain 1330, sequence accession CP002997 and CP002998 [6]
*Campylobacter fetus subsp. ven*erealis NCTC 10354, sequence accession AFGH01000000 [7]
*“Chromobacterium* sp.*”* strain C-61, sequence accession CAEE01000001 to CAEE01001118 [8]
*Cronobacter sakazakii* strain E899, sequence accession AFMO00000000 [9]
“*Desulfovibrio* sp.” strain A2, sequence accession AGFG01000000 [10]
“*Erythrobacter* sp.” strain NAP1, sequence accession NZ_AAMW00000000 [11]
*Escherichia coli* strain XH140A, sequence accession AFVX01000000 [12]
*Escherichia coli* strain XH001, sequence accession AFYG01000000 [13]
*Haemophilus haemolyticus* strain M19107, sequence accession AFQN00000000 [14]
*Haemophilus haemolyticus* strain M19501, sequence accession AFQO00000000 [14]
*Haemophilus haemolyticus* strain M21127, sequence accession AFQP00000000 [14]
*Haemophilus haemolyticus* strain M21621, sequence accession AFQQ00000000 [14]
*Haemophilus haemolyticus* strain M21639, sequence accession AFQR00000000 [14]
*Idiomarina* sp.” strain A28L, sequence accession AFPO01000001 to AFPO01000028 [15]
*Ketogulonicigenium vulgare*” strain WSH-001, sequence accession CP002018 (chromosome), CP002019 (plasmid pKVU_100), and CP002020 (plasmid pKVU_200) [16]
*Methylobacter tundripaludum* strain SV96, sequence accession  AEGW00000000 [17]
*Pseudogulbenkiania* *sp.*” strain NH8B, sequence accession AP012224 [18]
*Pseudomonas aeruginosa* NCGM1179, sequence accession DF126593 through DF126613 [19]
*Pseudomonas putida* strain B001, sequence accession CAED01000001 to CAEE01000262 [20]
*Pseudomonas putida* strain B6-2, sequence accession AGCS01000000 [21]
*Pseudomonas stutzeri* CGMCC 1.1803, sequence accession CP002881 [22]
*Ralstonia solanacearum* phylotype IB, strain Y45, sequence accession AFWL01000000 [23]
*Rheinheimera* sp.” strain A13L, sequence accession AFHI01000001 through AFHI01000072 [24]
*Sphingobium yanoikuyae* strain XLDN2-5, sequence accession AFXE01000000 [25]
*Vibrio cholerae* strain Amazonia, sequence accession AFSV01000000 [26]
**Phylum** *Firmicutes*
*Bacillus coagulans* strain XZL4, sequence accession AFWM01000000 [27]
*Bacillus megaterium* strain WSH-002, sequence accession CP003017 (chromosome), plasmids CP003018 (plasmid pBME_100), CP003019 (plasmid pBME_200), and CP003020 (plasmid pBME_300) [28]
*Bacillus pumilus* strain S-1, sequence accession AGBY00000000 [29]
*“Desulfosporosinus* sp.*”* strain OT, sequence accession AGAF01000000 [30]
*Lentibacillus jeotgali* strain Grbi, sequence accession AGAV01000000 [31]
*Leuconostoc carnosum* KCTC 3525, sequence accession BACM01000000 [32]
*Listeria ivanovii subsp. ivanovii* strain PAM 55, sequence accession FR687253 [33]
*Paenibacillus riograndensis* strain SBR5, sequence accession AGBD01000000 [34]
*Sporolactobacillus inulinus* strain CASD, sequence accession AFVQ00000000 [35]
*Streptococcus pseudopneumoniae* strain IS7493, sequence accession CP002925 and CP002926 [36]
*Streptococcus salivarius* strain 57.I, sequence accession CP002888 and CP002889 [37]
*Streptococcus salivarius* strain M18, sequence accession AGBV01000000 [38]
*Streptococcus suis* SS12, sequence accession CP002640 [39]
*Streptococcus suis* D9, sequence accession CP002641 [39]
*Streptococcus suis* D12, sequence accession CP002644 [39]
*Streptococcus suis* ST1, sequence accession CP002651 [39]
*Weissella thailandensis* strain fsh4-2, sequence accession HE575133 through HE575182 [40]
**Phylum** *Tenericutes*
*Mycoplasma anatis* strain 1340, sequence accession AFVJ00000000 [41]
*Mycoplasma capricolum subsp. capripneumoniae* strain M1601, sequence accession AENG01000000 [42]
*Mycoplasma putrefaciens* Type strain KS1, sequence accession CP003021 [43]
*Corynebacterium pseudotuberculosis* strain PAT10, sequence accession CP002924 [44]
**Phylum** *Actinobacteria*
*Bifidobacterium animalis subsp.* *lactis* strain BLC1, sequence accession CP003039 [45]
*Bifidobacterium breve* strain DPC 6330, sequence accession AFXX01000000 [46]
*Brachybacterium squillarum* strain M-6-3, sequence accession AGBX01000000 [47]
“*Citricoccus* sp.” strain CH26A, sequence accession AFXQ01000000 [48]
*Corynebacterium glutamicum* strain S9114, sequence accession AFYA01000000 [49]
*Dietzia alimentaria* strain 72, sequence accession AGFF01000000 [50]
*Mycobacterium colombiense* CECT 3035, sequence accession AFVW00000000 [51]
*Mycobacterium tuberculosis* NCGM2209, sequence accession DF126614 and DF126615 [52]
*Rhodococcus erythropolis* strain XP, sequence accession AGCF01000000 [53]
*Serinicoccus profundi* MCCC 1A05965^T^, sequence accession AFYF00000000 [54]
**Phylum** *Spirochaetes*
Leptospira interrogans, sequence accession CP001221 (CI), CP001222 (CII) [55]
**Phylum** *Bacteroidetes*
*Bacteroides faecis* Type strain MAJ27^T^, sequence accession AGDG01000000 [56]
*Bizionia argentinensis*, Type strain JUB59^T^ sequence accession AFXZ01000000 [57]
*Flavobacterium branchiophilum* strain FL-15, sequence accession FQ859183 [58]
*“Flavobacteriaceae”* strain S85, sequence accession AFPK00000000 [59]
**Phylum** *Thermotogae*
*“Thermotoga sp.”* strain RQ2, sequence accession CP000969 [60]

**Non-Bacterial genomes**
*Aspergillus kawachii* IFO 4308, sequence accession DF126447 through DF126592, BACL01000001 through BACL01001641, AP012272 [61]
*Cajanus cajan* pigeonpea, sequence accession PRJNA72815 [62]
Coxsackievirus A22, sequence accession JN542510 [63]
Gordonia phage GRU1, sequence accession JF923797 [64]
Gordonia phage GTE5, sequence accession JF923796 [64]
*Heterocephalus glaber* naked mole rat, sequence accession AFSB00000000, AFSB01000000 [65]
Human Adenovirus Prototype 17, sequence accession HQ910407 [66]
*Macaca mulatta lasiota* rhesus macaque, sequence accession AEHL00000000 [67]
*Macaca mulatta mulatta* rhesus macaque, sequence accession AEHK00000000 [67]
Porcine epidemic diarrhea virus, sequence accession JN547228 [68]
